# TRAJECTORIES OF PHENOTYPIC MARKERS OF AGING AS PRECURSORS TO FUNCTIONAL CHANGE

**DOI:** 10.1093/geroni/igz038.2146

**Published:** 2019-11-08

**Authors:** Jennifer A Schrack, Pei-Lun Kuo, Eleanor M Simonsick, Susan M Resnick, Morgan Levine, Michelle Shardell

**Affiliations:** 1 Johns Hopkins University, Baltimore, Maryland, United States; 2 National Institute on Aging, Baltimore, Maryland, United States; 3 Yale University, New Haven, Connecticut, United States

## Abstract

Delineating trajectories of aging phenotypes is essential to understanding mechanisms of clinical disease and disability. We investigated longitudinal changes in measures of body composition, energy expenditure, and brain volumes in >900 participants (age 67.0 (IQR: 57-77) years, 48.1% male) of the Baltimore Longitudinal Study of Aging using mixed effects regression models. Computed tomography-derived thigh muscle cross-sectional area declined 754.2 cm2 per decade at age 60 years (p<0.001) and 1294.3 cm2 at 75 years (p<0.001). Energy reserves, defined as a ratio of energy-cost-to-energy-capacity measured using indirect calorimetry, decreased 11.2% per decade at 60 years (p<0.001), and 16.8% at 75 years (p<0.001). MRI-derived measures of total brain volumes declined 41.6 cm3 per decade at 60 years (p<0.001) and 44.9 cm3 at 75 years (p<0.001). Linking these findings to biological and clinical measures of aging may contribute to more accurate assessment of phenotypic age.

